# Classical swine fever virus inhibits serine metabolism-mediated antiviral immunity by deacetylating modified PHGDH

**DOI:** 10.1128/mbio.02097-24

**Published:** 2024-08-29

**Authors:** Xiaowen Li, Yaoyao Huang, Xueyi Liu, Lihong Zhang, Xinyan Wang, Feifan Zhao, Linke Zou, Keke Wu, Wenxian Chen, Yuwei Qin, Sen Zeng, Bingke Li, Yintao He, Yiwan Song, Zhaoyao Li, Jindai Fan, Mingqiu Zhao, Lin Yi, Hongxing Ding, Shuangqi Fan, Jinding Chen

**Affiliations:** 1 College of Veterinary Medicine, South China Agricultural University, Guangzhou, China; College of Veterinary Medicine, Cornell University, Ithaca, New York, USA

**Keywords:** classical swine fever virus, serine metabolism, PHGDH, acetylation, innate immunity

## Abstract

Classical swine fever virus (CSFV), an obligate intracellular pathogen, hijacks cellular metabolism to evade immune surveillance and facilitate its replication. The precise mechanisms by which CSFV modulates immune metabolism remain largely unknown. Our study reveals that CSFV infection disrupts serine metabolism, which plays a crucial role in antiviral immunity. Notably, we discovered that CSFV infection leads to the deacetylation of PHGDH, a key enzyme in serine metabolism, resulting in autophagic degradation. This deacetylation impairs PHGDH’s enzymatic activity, reduces serine biosynthesis, weakens innate immunity, and promotes viral proliferation. Molecularly, CSFV infection induces the association of HDAC3 with PHGDH, leading to deacetylation at the K364 site. This modification attracts the E3 ubiquitin ligase RNF125, which facilitates the addition of K63-linked ubiquitin chains to PHGDH-K364R. Subsequently, PHGDH is targeted for lysosomal degradation by p62 and NDP52. Furthermore, the deacetylation of PHGDH disrupts its interaction with the NAD^+^ substrate, destabilizing the PHGDH-NAD complex, impeding the active site, and thereby inhibiting *de novo* serine synthesis. Additionally, our research indicates that deacetylated PHGDH suppresses the mitochondria-MAVS-IRF3 pathway through its regulatory effect on serine metabolism, leading to decreased IFN-β production and enhanced viral replication. Overall, our findings elucidate the complex interplay between CSFV and serine metabolism, revealing a novel aspect of viral immune evasion through the lens of immune metabolism.

**IMPORTANCE:**

Classical swine fever (CSF) seriously restricts the healthy development of China’s aquaculture industry, and the unclear pathogenic mechanism and pathogenesis of classical swine fever virus (CSFV) are the main obstacle to CSF prevention, control, and purification. Therefore, it is of great significance to explore the molecular mechanism of CSFV and host interplay, to search for the key signaling pathways and target molecules in the host that regulate the replication of CSFV infection, and to elucidate the mechanism of action of host immune dysfunction and immune escape due to CSFV infection for the development of novel CSFV vaccines and drugs. This study reveals the mechanism of serine metabolizing enzyme post-translational modifications and antiviral signaling proteins in the replication of CSFV and enriches the knowledge of CSFV infection and immune metabolism.

## INTRODUCTION

Classical swine fever (CSF) is an infectious disease characterized by immune escape and metabolic disorders caused by the classical swine fever virus (CSFV). CSF is prevalent worldwide, affecting regions such as Asia, Africa, Central America, South America, and Europe, leading to significant economic losses in the swine industry (1). Given its significant economic impact, outbreaks of CSF must be reported to the World Organization for Animal Health. CSFV belongs to the *Pestivirus* genus of the *Flaviviridae* family and is a small enveloped virus with a 12.3-kb positive-sense single-stranded RNA genome. CSFV can persist in pigs, leading to chronic infection. Despite the presence of specific antibodies in the body at this stage, the virus can still evade clearance by the host. This demonstrates the ability of CSFV to evade host immunity (2). Viruses use various mechanisms, including immune metabolism, to evade host immune clearance. Lipid and glucose metabolism are known to be involved in the activation of the innate immune response to regulate viral infections (3–6). For example, the infection of CSFV can change lipid metabolites, leading to the accumulation of free fatty acids and impairing type I IFN production, thereby facilitating continuous viral replication (3). Similarly, CSFV infection reduces LDHB-mediated glycolysis and inhibits NF-κB signaling pathway activation through LDHB-induced mitophagy (5). However, the role of amino acid metabolism in CSFV immune evasion remains largely unknown.

Serine metabolism not only provides precursors for tumor cell growth to synthesize nucleic acids, proteins, and lipids but also participates in the activation of innate immunity. Serine is converted to glycine, which promotes one-carbon metabolism, supports nucleotide synthesis, and enhances the proliferation of cancer cells and T cells (7–10). Furthermore, a previous study showed that GSH, a serine metabolite, is essential for maintaining lipopolysaccharide-induced IL-1β production (11). Moreover, serine metabolism promotes lipopolysaccharide-induced inflammation through S-adenosylmethionine (SAM)-mediated histone methylation in macrophages (12). For macrophage inflammation, serine metabolism can be mediated by regulating IL-1β production and adjusting macrophage polarization. It promotes the polarization of interleukin-4-activated macrophages through SAM-mediated histone methylation (13). In addition, this metabolic pathway is involved in antiviral innate immunity through SAM-mediated histone methylation in macrophages, which enhances YAP-mediated inhibition of the TBK1–IRF3 axis (14). Therefore, how serine metabolism behaves under viral infection is an interesting question.

PHGDH is the rate-limiting enzyme in *de novo* serine biosynthesis and plays a crucial role in converting glycolysis-derived 3-phosphoglycerate into serine. In recent years, the importance of PHGDH in cancer is increasingly being recognized. Remarkably, PHGDH expression is significantly upregulated in a variety of cancers, including colorectal, gastric, bladder, breast, and lung cancers (15–19). One recent study shows that the high expression of PHGDH is closely related to the low expression of Parkin in both breast and lung cancers (18). Parkin, an E3 ubiquitin ligase, mediates PHGDH ubiquitination and degradation, inhibiting serine synthesis, cell proliferation, and tumorigenesis (18). In colorectal cancer, PHGDH undergoes monoubiquitylation by cullin 4A, which enhances its activity and promotes tumor cell migration and colorectal cancer metastasis through SAM-mediated histone methylation (19). The interplay between PHGDH and viral infections has also been studied. A recent report has shown that PHGDH is downregulated during viral infections (14). However, whether post-translational modification affects the expression of PHGDH in virus-infected cells and how the downregulation of PHGDH is involved in viral replication remains unknown.

Recent studies have indicated that protein function can be co-ordinately regulated by acetylation and ubiquitination. Some metabolic enzymes have been found to regulate protein stability through acetylation and ubiquitination synergistically. Acetylation regulates protein degradation, such as ubiquitin-dependent proteasomal degradation or lysosome-dependent degradation (20, 21). We report that CSFV infection leads to the downregulation of serine metabolism and inhibits serine metabolism-mediated antiviral immunity. Molecularly, CSFV infection induces deacetylation modification of PHGDH, reducing PHGDH enzyme activity and subsequent autophagic lysosomal degradation. In addition, CSFV inhibits IFN-β production by decreasing PHGDH expression, thereby impeding PHGDH-mediated binding of RIG-I and MAVS. Our findings reveal a novel molecular mechanism that the acetylation of PHGDH in serine metabolism regulates innate immunity after CSFV infection.

## RESULTS

### CSFV infection inhibits serine metabolism

**Fig 1:**
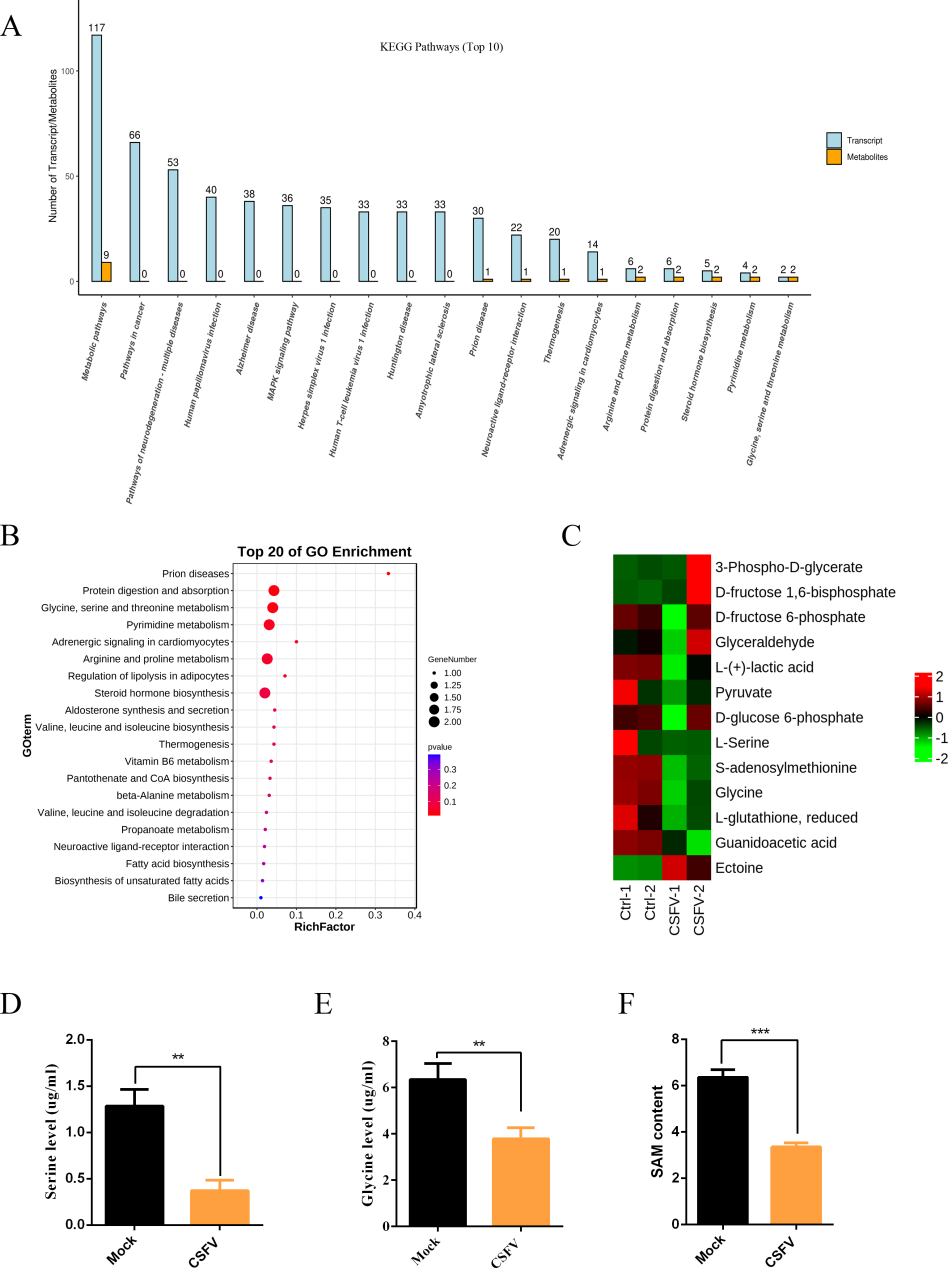
CSFV infection inhibits serine metabolism. (**A**) Combined metabolomics and transcriptomics analysis of signaling pathways underlying significant changes in CSFV-infected PAMs. (**B**) KEGG analysis of significantly altered metabolic pathways in CSFV-infected PAMs. (**C**) Significantly altered metabolites in CSFV-infected PAMs. (**D**) Determination of serine in CSFV-infected cells by liquid chromatography. (**E**) Determination of glycine in CSFV-infected cells by liquid chromatography. (**F**) ELISA to determine SAM content in CSFV-infected cells. For (D–F), error bars indicate the mean [±standard deviation (SD)] of three independent experiments. ^**^P < 0.01 and ^***^P < 0.001 (*t*-tests).

To investigate the pathogenic mechanism of CSFV, we conducted a joint transcriptomic and metabolomic analysis on alveolar macrophages infected with CSFV and those uninfected. The results revealed that CSFV infection leads to amino acid metabolic disorders, such as disruptions in arginine and proline metabolism, as well as glycine–serine–threonine metabolism (Fig. 1A). Additionally, metabolomic KEGG analysis indicated that CSFV infection results in the downregulation of glycine–serine–threonine metabolism (Fig. 1B), causing reduced secretion levels of serine, glycine, and SAM (Fig. 1C). To further clarify the impact of CSFV on serine metabolism, we also measured the levels of serine metabolites in CSFV-infected cells using liquid chromatography and enzyme-linked immunosorbent assay (ELISA), revealing that CSFV infection leads to a decrease in the secretion levels of serine (Fig. 1D), glycine (Fig. 1E), and SAM (Fig. 1F).

### CSFV NS4A interacted with PHGDH and decreased PHGDH expression

**Fig 2:**
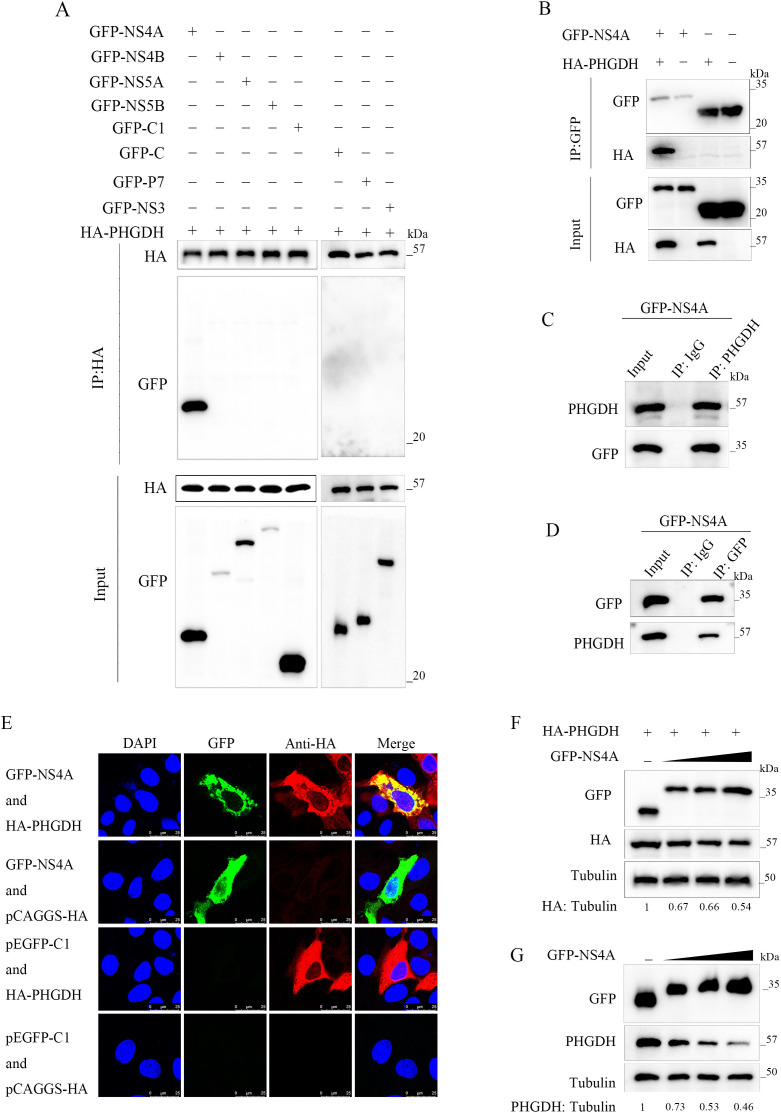
CSFV NS4A interacted with PHGDH and decreased PHGDH expression. (**A**) Screening of CSFV replication-associated proteins for interaction with PHGDH by the co-IP assay. (**B**) Validation of CSFV NS4A interaction with PHGDH by the co-IP assay. (**C and D**) Immunoprecipitation (IP) assay validates binding of CSFV NS4A to endogenous PHGDH. (**E**) Laser confocal observation of the localization of PHGDH and NS4A in cells. (**F**) Western blot to verify the effect of NS4A on exogenous PHGDH. (**G**) Western blot to verify the effect of NS4A on endogenous PHGDH.

To elucidate the direct link between CSFV infection and serine metabolism, we examined the interactions between serine metabolic enzymes PHGDH, PSAT1, and PSPH and CSFV viral proteins (C, p7, NS3, NS4A, NS4B, NS5A, and NS5B). Our findings revealed that only PHGDH was capable of interacting with the CSFV NS4A protein (Fig. 2A), while PSAT1 and PSPH did not exhibit any interaction with CSFV viral proteins (Fig. S1). Further co-immunoprecipitation (co-IP) experiments demonstrated that PHGDH binds with NS4A both intracellularly and extracellularly (Fig. 2B through D). Additionally, confocal microscopy revealed the co-localization of PHGDH with NS4A in the cytoplasm (Fig. 2E). To determine the impact of NS4A on PHGDH protein expression, we overexpressed NS4A exogenously. The results indicate that NS4A suppresses the protein expression levels of both exogenous and endogenous PHGDH, with a decrease correlating to the increased dosage of NS4A (Fig. 2F and G).

### CSFV inhibits the activity of the PHGDH protein and its acetylation modification

**Fig 3:**
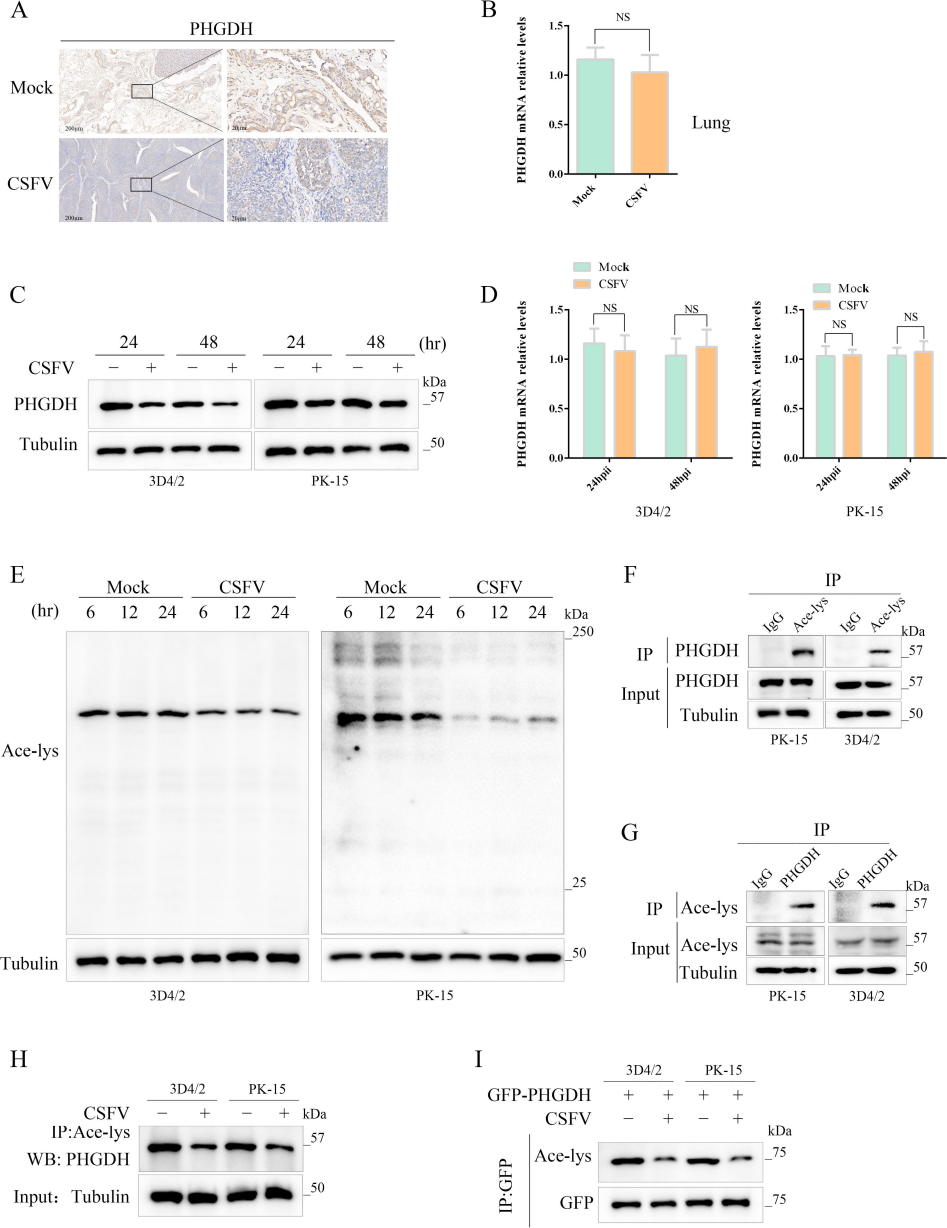
CSFV inhibits the activity of the PHGDH protein and its acetylation modification. (**A**) Immunohistochemical identification of PHGDH protein expression after CSFV infection of tissues. (**B**) qPCR detection of PHGDH mRNA transcript levels after CSFV infection of tissues. (**C**) Western blot detection of PHGDH protein expression in CSFV-infected PK-15 cells and 3D4/2. (**D**) qPCR detection of PHGDH mRNA transcript levels in CSFV-infected PK-15 cells and 3D4/2. (**E**) Western blot detection of the effect of CSFV infection on the acetylation level of cells. (**F**) IP experiments were performed with Ace-lys as a specific antibody to determine the binding of PHGDH to Ace-lys. (**G**) The binding of PHGDH to Ace-lys was determined in IP experiments using PHGDH as a specific antibody. (**H**) Characterization of CSFV effects on PHGDH acetylation by IP assay. (**I**) Characterization of CSFV effects on PHGDH acetylation by the co-IP assay.

To further explore the association between CSFV infection and PHGDH expression, we utilized immunohistochemistry and quantitative real-time RT-PCR (qPCR) to detect PHGDH expression in CSFV-infected tissues. The findings revealed that CSFV infection significantly reduced PHGDH expression in swine tissues (Fig. 3A), although there was no significant difference in PHGDH mRNA transcription levels in the CSFV-infected tissues (Fig. 3B). To further assess the effects of CSFV infection on PHGDH, we infected PK-15 cells and 3D4/2 cells with CSFV. The results indicated that CSFV infection led to a decrease in PHGDH protein expression levels at 24 and 48 h post-infection in both PK-15 and 3D4/2 cells (Fig. 3C), while there was no significant impact on PHGDH mRNA transcription levels (Fig. 3D). Moreover, we investigated the impact of CSFV infection on PHGDH enzyme activity. The results showed that CSFV infection in PK-15 and 3D4/2 cells led to a reduction in PHGDH enzyme activity (Fig. S2). These outcomes suggest that the reduction in PHGDH protein levels due to CSFV infection is not attributable to changes in mRNA but is likely due to post-translational modifications of the protein. Post-translational modifications can endow proteins with functional diversity. To probe the influence of CSFV infection on cellular acetylation levels, we discovered that CSFV infection led to a decrease in overall cellular acetylation levels (Fig. 3E). Acetylation regulates cellular metabolome and modulates the activity of several metabolic enzymes upon cellular stimulation (20, 22, 23). To delve into whether PHGDH is a substrate for acetylation modification, we examined the acetylation levels of PHGDH in PK-15 and 3D4/2 cells. After immunoprecipitation (IP) assays with anti-Ace-lys and anti-PHGDH, we discovered that Ace-lys could enrich PHGDH, indicating that PHGDH is a substrate for Ace-lys (Fig. 3F and G). Our findings corroborate previous reports on the acetylation of PHGDH (24). Continuing our exploration of the effects of CSFV infection on PHGDH acetylation, we found that CSFV infection in PK-15 and 3D4/2 cells led to a decrease in PHGDH acetylation levels (Fig. 3H and I), suggesting that CSFV mediates the deacetylation modification of PHGDH.

### Histone acetyltransferase KAT8 interacts with PHGDH via its MYST domain

**Fig 4:**
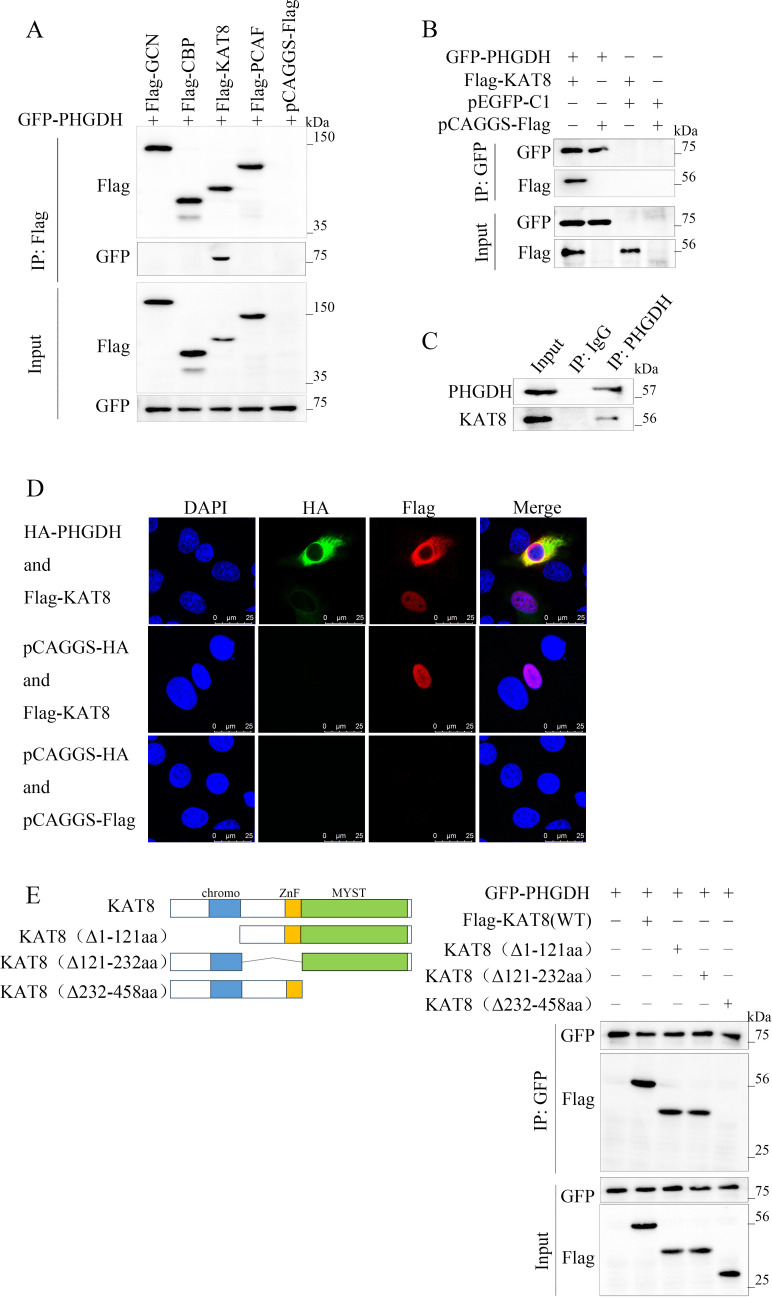
Histone acetyltransferase KAT8 interacts with PHGDH via its MYST domain. (**A**) Co-immunoprecipitation of exogenous PHGDH and acetyltransferases in PK-15 cells. The acetyltransferases were precipitated with anti-Flag, and the precipitates were probed with anti-GFP. (**B**) PHGDH interacts with KAT8. Western blot analysis of the interaction between ectopic PHGDH and KAT8 in PK-15 cells. (**C**) Immunoprecipitation assay for the interaction between endogenous PHGDH and KAT8. (**D**) Laser confocal microscopy of PHGDH and KAT8 localization in PK-15 cells. (**E**) Identification of the structural domain of KAT8 binding to PHGDH.

Protein acetylation modifications are regulated by acetyltransferases. To further discover the acetyltransferases that regulate the acetylation modification of PHGDH, we expressed several common acetyltransferases, GCN, CBP, KAT8, and PCAF, respectively, in cells and examined their association with PHGDH. The results revealed that GFP-PHGDH formed a protein complex with Flag-KAT8 and did not interact with the other transferases (Fig. 4A). Further co-IP experiments confirmed that GFP-PHGDH formed a complex with Flag-KAT8, but not with pCAGGS-Flag (Fig. 4B). Additionally, PHGDH was found to form a protein complex with endogenous KAT8 by IP assays using a PHGDH-specific antibody (Fig. 4C). To further validate the interaction between PHGDH and KAT8, we observed their cellular localization through confocal laser microscopy. We noted that when KAT8 co-existed with PHGDH, KAT8 translocated from the nucleus to the cytoplasm and co-localized with PHGDH in the cytoplasm (Fig. 4D). To more clearly determine the region of PHGDH that binds to KAT8, we further constructed three different truncated versions of Flag-tagged KAT8, each lacking the chromatin domain (Δ1–121 aa), the C2HC zinc sheath domain (Δ121–232 aa), and the enzyme MYST domain (Δ232–458 aa). Among these truncations, only the one lacking the MYST domain failed to interact with PHGDH (Fig. 4E). Therefore, the MYST domain of KAT8 is responsible for its interaction with PHGDH. These results suggest that KAT8 directly binds to PHGDH through its acetyltransferase activity within the MYST domain.

### PHGDH is acetylated by KAT8 at lysine 364

**Fig 5:**
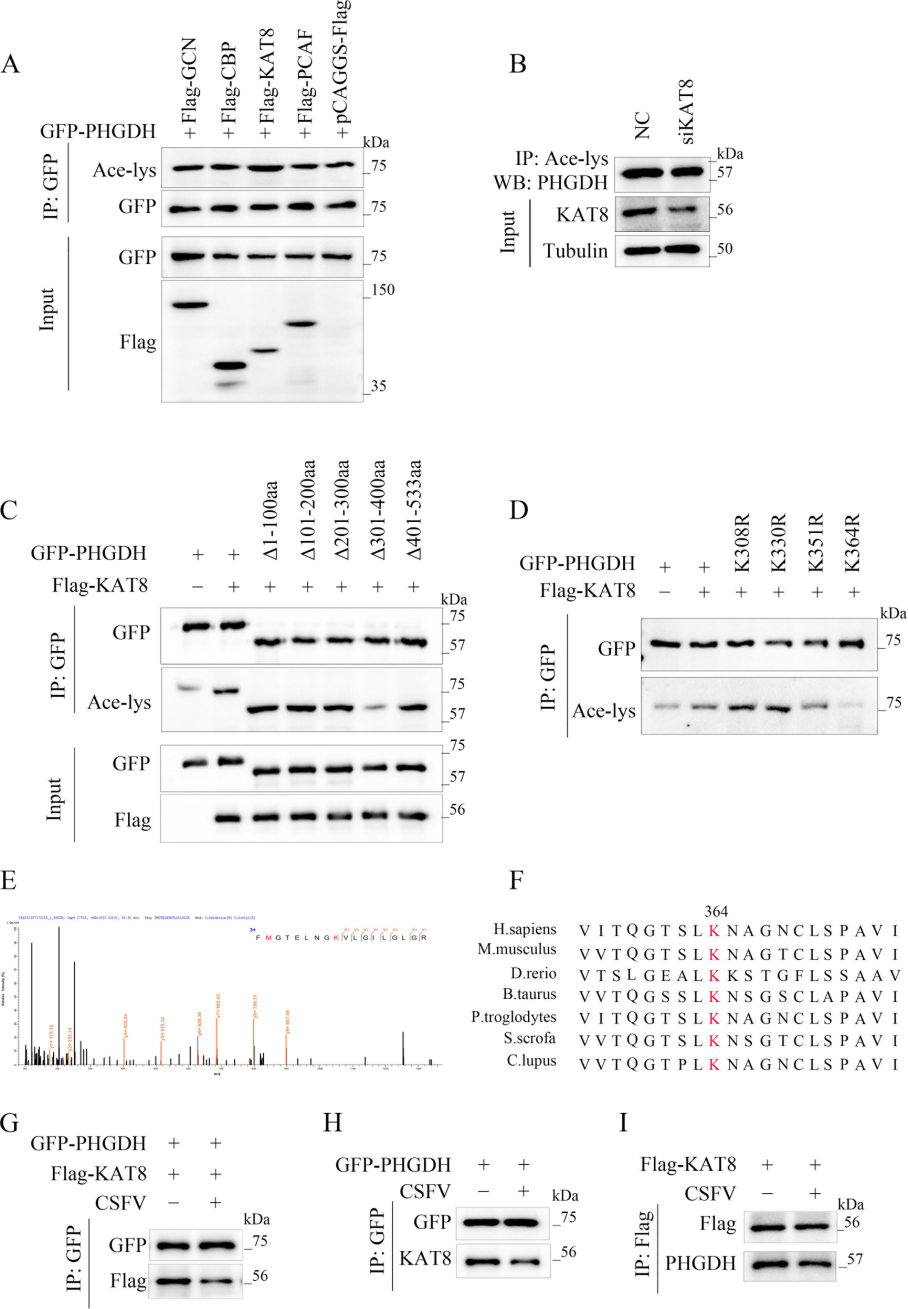
PHGDH is acetylated by KAT8 at lysine 364. (**A**) PHGDH acetylation in PK-15 cells expressing various acetylases. (**B**) PHGDH acetylation in PK-15 cells with KAT8 knockdown. (**C**) PHGDH acetylation region. PHGDH acetylation analyzed by immunoblotting in PK-15 cells expressing different truncated mutants of GFP-PHGDH (every 100 amino acids) and Flag-KAT8. (**D**) Acetylation of GFP-PHGDH or its mutants in PK-15 cells assessed by immunoblotting. (**E**) Mass spectrometry analysis of PHGDH acetylation sites in PK-15 cells. (**F**) Amino acid sequence alignment of PHGDH from different species. Red shading indicates conserved K364. (**G, H, and I**) co-IP and IP assays characterize the effect of CSFV infection on the binding of PHGDH to KAT8.

KAT8 possesses acetyltransferase activity, and to further investigate whether PHGDH is an acetylation substrate of KAT8, we co-expressed various common acetyltransferases in PK-15 cells. We discovered that the acetyltransferase KAT8, not the others, significantly enhanced the acetylation level of PHGDH (Fig. 5A). Additionally, silencing the expression of the KAT8 gene in PK-15 cells led to a decrease in the basal acetylation level of PHGDH (Fig. 5B). These results indicated that KAT8 could regulate the acetylation modification of PHGDH. To further identify the potential acetylated lysine residues of PHGDH, we constructed a series of PHGDH truncations and found that the truncation missing amino acids 301–400 weakened the KAT8-mediated acetylation of PHGDH (Fig. 5C), suggesting that the acetylation site is within amino acids 301–400. This region contains four lysine residues, and we mutated each lysine to arginine to identify the acetylation sites. Compared to other mutants, K364R completely lost acetylation, indicating that lysine at position 364 is the primary acetylation site of PHGDH (Fig. 5D). Additionally, mass spectrometry analysis confirmed the acetylation of lysine at position 364 of PHGDH (Fig. 5E), and this site is highly conserved across species (Fig. 5F). The above results suggest that KAT8 regulates the PHGDH K364 site to undergo acetylation modification. Further exploring the impact of CSFV infection on KAT8-mediated PHGDH acetylation modification, we found that CSFV infection reduced the interaction between KAT8 and PHGDH (Fig. 5G through I).

### CSFV utilize HDAC3 to promote PHGDH deacetylation

**Fig 6:**
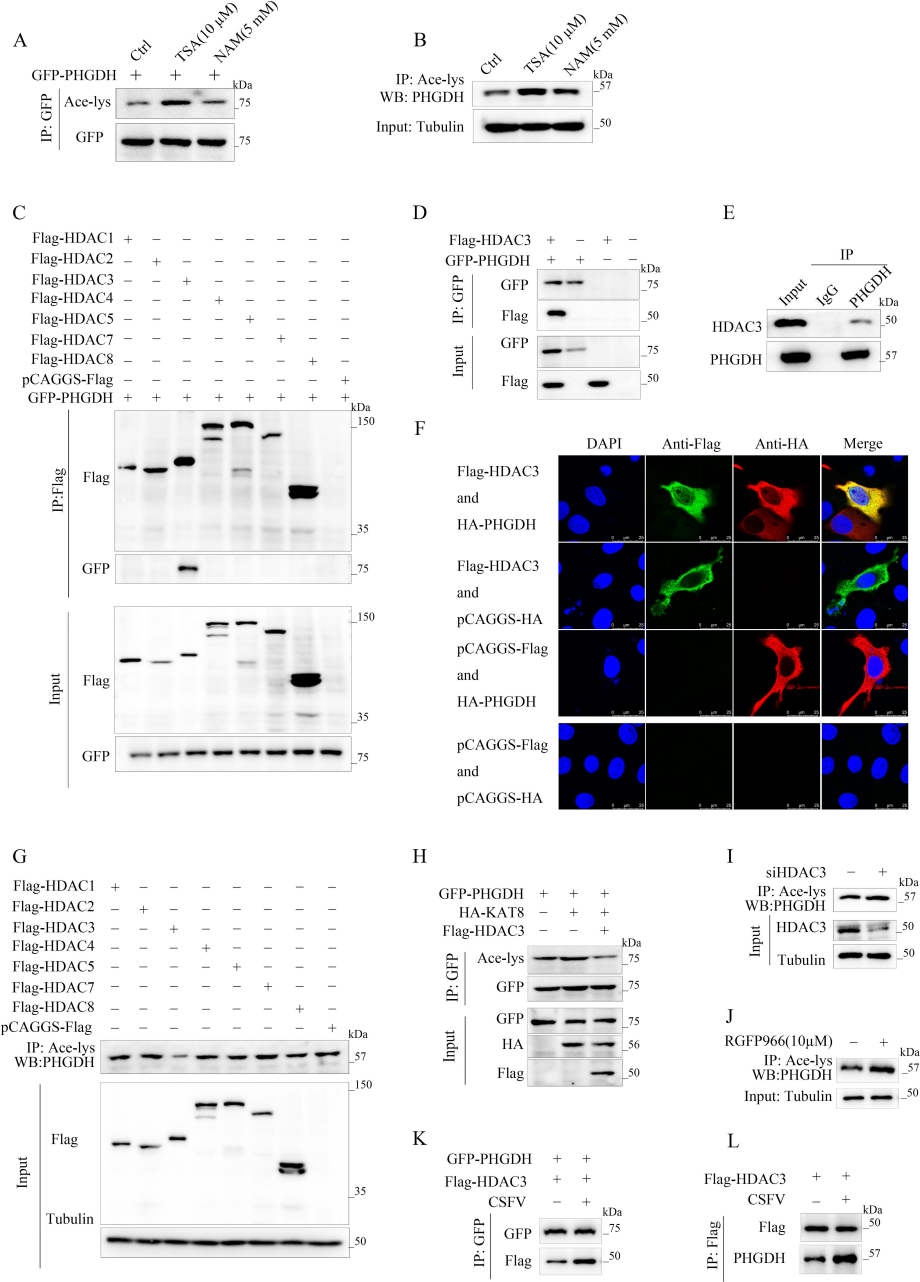
CSFV utilize HDAC3 to promote PHGDH deacetylation. (**A and B**) PHGDH acetylation in PK-15 cells treated with TSA (10 µM) and NAM (5 mM) detected by immunoblotting. (**C**) Co-immunoprecipitation of exogenous PHGDH and deacetyltransferases in PK-15 cells. The deacetyltransferases were precipitated with anti-Flag, and the precipitates were probed with anti-GFP. (**D**) PHGDH interacts with HDAC3. Western blot analysis of the interaction between ectopic PHGDH and HDAC3 in PK-15 cells. (**E**) Immunoprecipitation analysis of endogenous PHGDH interaction with HDAC3. (**F**) Laser confocal microscopy of PHGDH and HDAC3 localization in PK-15 cells. (**G**) PHGDH acetylation in PK-15 cells expressing various deacetylases. (**H**) PHGDH acetylation in PK-15 cells with or without HA-KAT8 and Flag-HDAC3 expression, assessed by immunoblotting. (**I**) PHGDH acetylation in PK-15 cells with HDAC3 knockdown. (**J**) PHGDH acetylation in PK-15 cells treated with RGFP966 (10 µM). (**K and L**) Effect of CSFV infection on PHGDH binding to HDAC3 detected by the co-IP assay.

Previous studies have shown that several metabolic enzymes are regulated by the sirtuin family inhibitor nicotinamide (NAM) and HDAC family inhibitor trichostatin A (TSA) (22, 25, 26). To detect which family regulates the acetylation of PHGDH, we analyzed the acetylation of exogenously expressed PHGDH (Fig. 6A) and endogenous PHGDH (Fig. 6B) in cells treated with 10 µM TSA and 5 mM NAM. In our study, we observed that cells treated with TSA exhibited a markedly higher level of acetylation compared to those treated with NAM (Fig. 6A and B). The effect of TSA rather than NAM indicates that HDACs are involved in PHGDH deacetylation. We then tested the possible interaction between PHGDH and each of the HDACs and found that PHGDH interacted strongly and specifically with HDAC3, a metabolic deacetylase, in PK-15 cells (Fig. 6C through E). We confirmed the interaction between HDAC3 and PHGDH in cells by co-transfecting Flag-HDAC3 and HA-PHGDH in PK-15 cells and observing that HDAC3 co-localized with PHGDH in the cytoplasm (Fig. 6F). In addition, we overexpressed HDAC3 or other HDACs in cells and observed that only HDAC3 overexpression markedly reduced PHGDH acetylation (Fig. 6G). Importantly, HDAC3 overexpression also abolished KAT8-mediated PHGDH acetylation (Fig. 6H), suggesting that HDAC3 is the main PHGDH deacetylase. To further investigate the deacetylation of endogenous PHGDH by HDAC3, we knocked down HDAC3 in PK-15 cells and detected increased PHGDH acetylation (Fig. 6I). We also used RGFP966, a selective HDAC3 inhibitor (27), and found that RGFP966 treatment significantly increased the basal acetylation level of PHGDH (Fig. 6J). These results demonstrate that HDAC3 is the key deacetylase of PHGDH. To further investigate whether CSFV regulates the deacetylation of PHGDH via HDAC3, our findings indicate that CSFV infection facilitates the interaction between HDAC3 and PHGDH (Fig. 6K and L).

### Deacetylation of K364 facilitates PHGDH degradation by autophagy

**Fig 7:**
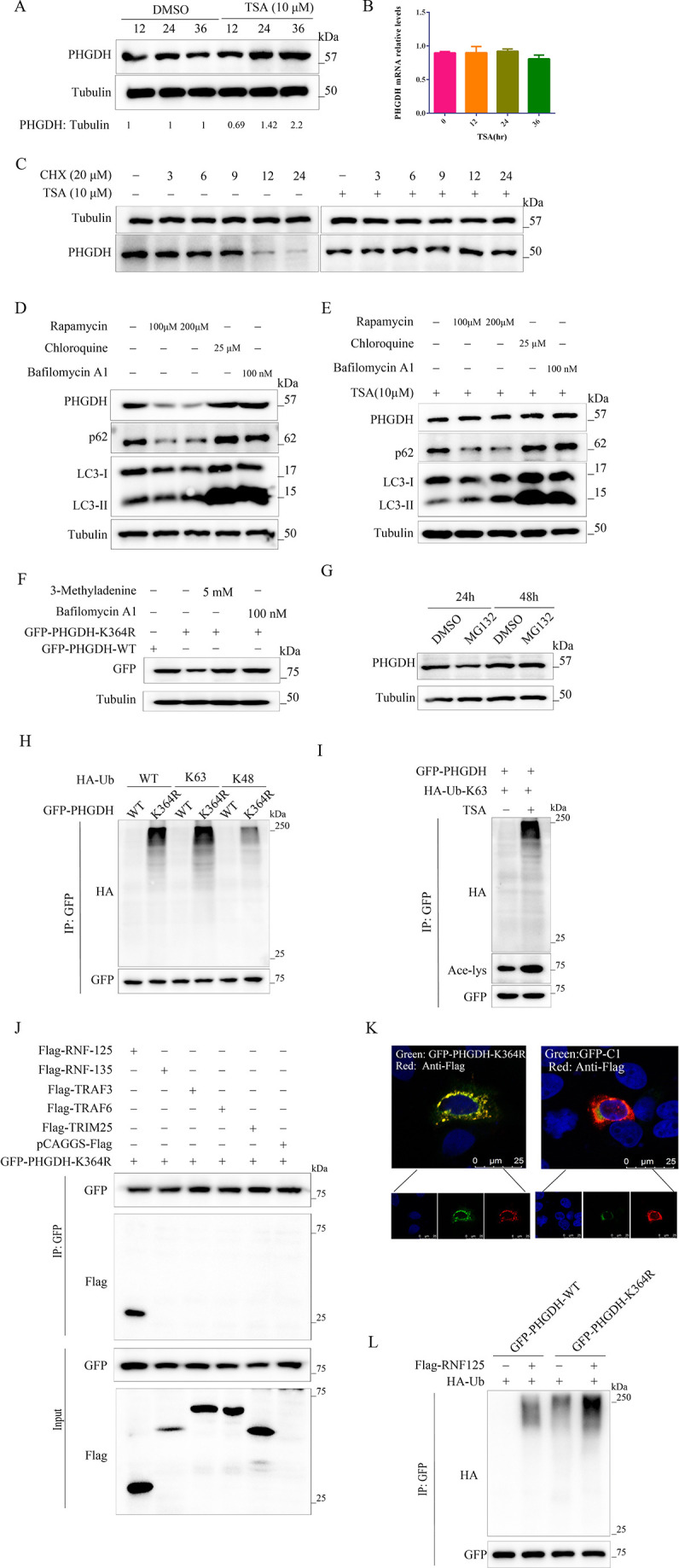
Deacetylation of K364 facilitates PHGDH degradation by autophagy. (**A**) PHGDH protein levels in PK-15 cells treated with TSA (10 µM) at different time points, detected by western blotting. (**B**) PHGDH mRNA levels in TSA-treated PK-15 cells, measured by qPCR. (**C**) PHGDH protein levels in PK-15 cells treated with TSA (10 µM) or CHX (20 µM) at different time points, assessed by western blotting. (**D**) PHGDH degradation by autophagic pathways. PK-15 cells were treated with the autophagy inducer rapamycin and the autophagy inhibitors chloroquine (25 µM) and bafilomycin A1 (100 mM). (**E**) PHGDH autophagic degradation inhibited by TSA. PK-15 cells were treated with the autophagy inducer rapamycin and the autophagy inhibitors chloroquine (25 µM), bafilomycin A1 (100 mM), and TSA (10 µM). (**F**) PHGDH degradation mediated by K364 deacetylation. PK-15 cells expressing PHGDH or its mutants were treated with 3-methyladenine (5 mM) and bafilomycin A1 (100 mM). (**G**) PHGDH protein levels in PK-15 cells treated with MG132 (10 µM) at different time points, quantified by western blotting. (**H**) PHGDH binding to the HA-Ub-63 ubiquitin chain enhanced by K364R mutation. PHGDH-WT and K364R were co-transfected into PK-15 cells with HA-Ub WT, K48-only, and K63-only mutants. (**I**) PHGDH and K63 poly-Ub chain association increased by TSA treatment. PK-15 cells co-transfected with PHGDH and HA-Ub-K63 were treated with TSA for 24 h. (**J**) Co-immunoprecipitation of exogenous PHGDH and E3 ubiquitin ligases in PK-15 cells. PHGDH was precipitated with anti-GFP, and the precipitates were analyzed with anti-Flag. (**K**) Laser confocal microscopy of PHGDH and RNF125 localization in PK-15 cells. (**L**) PHGDH ubiquitination augmented by RNF125. RNF125 was co-transfected with PHGDH or its mutant and HA-Ub in PK-15 cells.

Acetylation modulates protein stability, as previous studies have demonstrated. To investigate whether the protein stability of PHGDH is also affected by TSA, a deacetylase inhibitor, we treated cells with 10 µM TSA and observed a time-dependent accumulation of PHGDH protein (Fig. 7A). PHGDH mRNA levels remained unchanged after TSA treatment, as measured by qRT-PCR (Fig. 7B), ruling out the possibility that increased mRNA expression accounted for PHGDH protein accumulation. We then inhibited protein synthesis with 20 µM cycloheximide (CHX) and found that PHGDH was a stable protein in PK-15 cells, with a half-life of more than 9 h, but its degradation was impaired by TSA treatment (Fig. 7C). These results suggest that PHGDH protein accumulation is caused by reduced degradation due to acetylation, which is known to regulate protein degradation (28). Proteins with long half-lives are typically degraded by lysosomal autophagy (29, 30), so we treated cells with the autophagy inducer rapamycin or the autophagy inhibitors chloroquine and bafilomycin A1 (BafA1). Rapamycin enhanced the degradation of endogenous PHGDH, whereas chloroquine and BafA1 increased its accumulation (Fig. 7D), demonstrating that PHGDH degradation is mediated by lysosomal autophagy. Moreover, TSA treatment prevented the rapamycin-induced degradation of PHGDH (Fig. 7E). We also observed that deacetylation at K364 reduced the expression of exogenous PHGDH, and this effect was blocked by 3-methyladenine and BafA1 (Fig. 7F). To assess the role of the ubiquitin-proteasome pathway in PHGDH stability, we treated cells with the proteasome inhibitor MG132 (10 µM) and found no effect on PHGDH protein levels (Fig. 7G), indicating that PHGDH degradation is proteasome-independent.

Autophagy degrades cellular components in a nonselective or selective manner. Nonselective autophagy randomly sequesters cytoplasmic contents into phagophores, which mature into autophagosomes and fuse with lysosomes for cargo degradation. Selective autophagy, on the other hand, specifically targets proteins and organelles for degradation by autophagy receptors (31). Nonselective autophagy is mainly triggered by starvation, while selective autophagy serves various cellular functions, such as adaptation to environmental/nutritional changes and removal of damaged organelles (32). In selective autophagy, autophagy receptors, such as SQSTM1, NBR1, and CALCOCO2/NDP52, link their cytosolic substrates to MAP1LC3B (or related Atg8 family members) proteins on the phagophore membranes, thereby enabling cargo recognition (33). To uncover how PHGDH-K364R is degraded by autophagy, we first searched for the autophagy receptors of PHGDH-K364R. Among the four receptors, SQSTM1 and NDP52 bound to PHGDH-K364R (Fig. S3A through D). We then confirmed that SQSTM1 and NDP52 mediated the degradation of PHGDH by showing that PHGDH degradation was markedly impaired in SQSTM1-knockdown cells (Fig. S3E) or NDP52-knockdown cells (Fig. S3F). These results indicate that deacetylated PHGDH is degraded by autophagy. The protein requires ubiquitination before it is delivered to the autophagy-lysosome pathway for degradation by the autophagy receptor (34, 35). We next examined the ubiquitination of PHGDH by co-overexpressing GFP-PHGDH and HA-Ub in PK-15 cells. We found that the K364R mutant bound more ubiquitin than PHGDH-WT (Fig. 7H, lane 2), especially K63-linked ubiquitin (Fig. 7H, lane 4). Under normal conditions, PHGDH-WT barely interacted with ubiquitin, either K63-linked or K48-linked ubiquitin (Fig. 7H, lanes 1, 3, and 5). Increased acetylation due to TSA treatment also enhanced the association of PHGDH-WT with K63-linked polyubiquitin chains (Fig. 7I).

Ubiquitin E3 ligase is essential for the ubiquitin system (36). To identify the E3 ubiquitin ligase for PHGDH, we chose several common E3 ubiquitin ligases, such as RNF125, RNF135, TRAF3, TRAF6, and TRIM25. Among the chosen E3 ligases, only RNF125 interacted with PHGDH-K364R (Fig. 7J and K). RNF125 overexpression enhanced the ubiquitination of PHGDH, indicating that RNF125 is the E3 ubiquitin ligase of PHGDH (Fig. 7L, lane 2). RNF125 also increased the ubiquitination of the K364R mutant compared with PHGDH-WT (Fig. 7L, lane 4). These results imply that lysine 364 deacetylation facilitates PHGDH degradation via the autophagy pathway.

### Acetylation of K364 enhances the protein kinase activity of PHGDH

**Fig 8:**
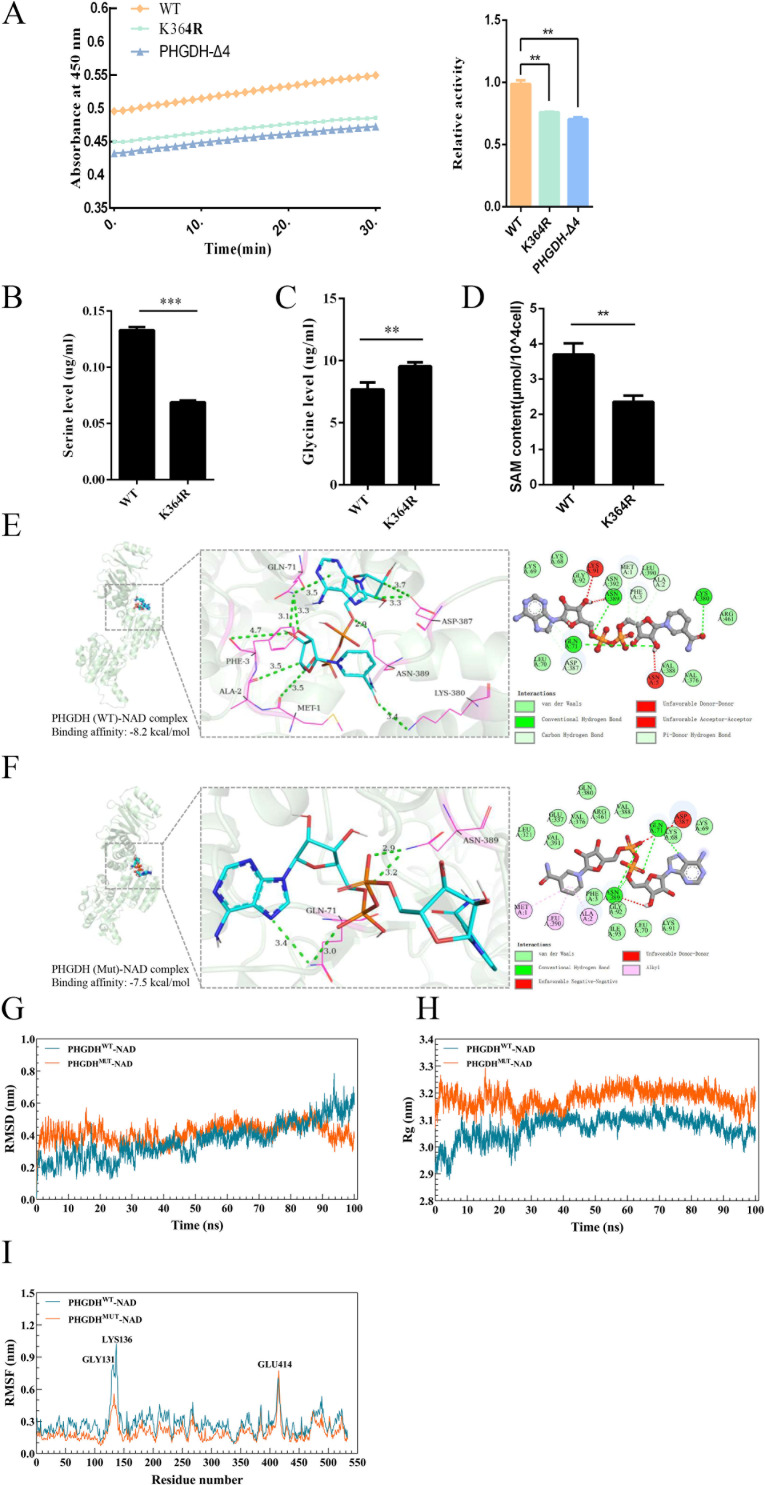
Acetylation of K364 enhances the protein kinase activity of PHGDH. (**A**) PHGDH enzyme activity impaired by K364 mutation. PHGDH activity in PK-15 cells expressing PHGDH or its mutants was measured by the kinetic model of the microplate reader. (**B**) PHGDH-K364R lowers serine levels. Serine levels in PK-15 cells expressing PHGDH and its mutants were assessed by liquid chromatography. (**C**) PHGDH-K364R raises glycine levels. Glycine levels in PK-15 cells expressing PHGDH and its mutants were evaluated by liquid chromatography. (**D**) PHGDH-K364R decreases SAM levels. SAM levels in PK-15 cells expressing PHGDH and its mutants were quantified by ELISA. (**E**) Molecular interaction of PHGDH-WT with its substrate (NAD^+^). (**F**) Molecular interaction of PHGDH mutants with its substrate (NAD^+^). (**G**) Root mean square deviation (RMSD) to evaluate the accuracy of protein structure prediction. (**H**) Radius of gyration (Rg), physical quantity used to describe the compactness of a protein’s structure. (**I**) Root mean square fluctuation (RMSF) measures particle mobility. For (A–D), error bars indicate the mean (±SD) of three independent experiments. ^**^P < 0.01 and ^***^P < 0.001 (*t*-tests).

PHGDH is an important metabolic enzyme that catalyzes serine synthesis. Therefore, it was hypothesized that HDAC3 deacetylation of PHGDH could modulate its enzymatic activity. To determine the effect of K364 deacetylation on the enzymatic activity of PHGDH, we employed the ELISA method to measure the enzymatic activity of PHGDH-WT, PHGDH-K364R, and PHGDH-Δ4. As shown in Fig. 8A, it was found that the enzyme activities of PHGDH-K364R and PHGDH-Δ4 were reduced relative to those of PHGDH-WT in PK-15 cells, which suggests that HDAC3-mediated deacetylation inactivates PHGDH to some extent. PHGDH is the rate-limiting enzyme for serine synthesis, so we examined the impact of PHGDH-K364R on serine synthesis and found that PHGDH-K364R-expressing cells had lower serine synthesis but higher glycine synthesis than PHGDH-WT cells (Fig. 8B and C). SAM, a crucial product of serine metabolism that modulates cellular immunity, was also significantly decreased by the K364R mutant compared with the control (Fig. 8D). These results imply that deacetylation of PHGDH diminishes its enzymatic activity and suppresses serine synthesis *de novo*.

To investigate the underlying structural mechanism of acetylation-induced changes in enzyme activity, we established homology models of wild-type PHGDH^WT^ and its acetylation site mutant PHGDH^Mut^ with the substrate NAD^+^ and molecularly docked the proteins and small molecules to analyze the degree of binding of PHGDH^WT^ and its PHGDH^Mut^ to the substrate NAD^+^ by CB-Dock2 software (Fig. S4). It was found that although PHGDH^Mut^ induced only subtle conformational changes, they led to different binding conformations of the substrate. Compared to PHGDH^WT^, PHGDH^Mut^ became less hydrogen-bonded to its substrate NAD^+^ (Fig. 8E and F). As shown in Fig. 8E, NAD^+^ was able to embed well into the active center of PHGDH^WT^ and interact with the surrounding amino acid residues with better affinity (−8.2 kcal/mol), whereas NAD^+^ required a stronger affinity (−7.5 kcal/mol) to be chimeric with PHGDH^Mut^ (Fig. 8F). The results suggest that the acetylation site of the mutant PHGDH leads to a reduction in its binding to the substrate, thus preventing the *de novo* synthesis of serine. Additionally, the molecular dynamic simulations of the wild-type PHGDH and its K364R mutants show that K364R has fewer hydrogen bond interactions with its binding partner, resulting in reduced complex stability (Fig. 8G through I). All of the above molecular docking and molecular dynamics simulations indicate that PHGDH deacetylation decreases its substrate binding and, thus, inhibits serine synthesis.

### PHGDH-mediated serine metabolism enhances cellular antiviral response to IFN-β

**Fig 9:**
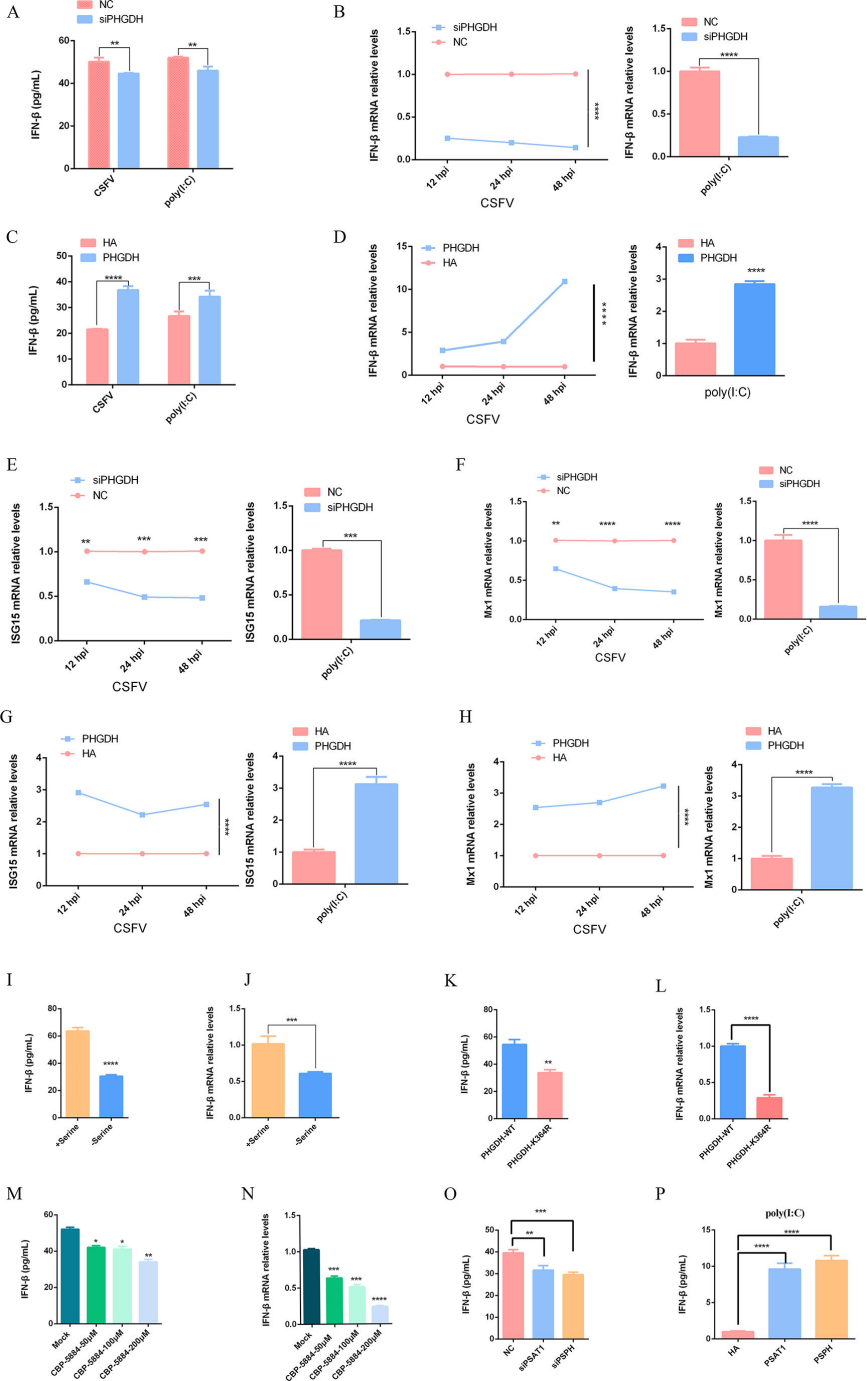
PHGDH-mediated serine metabolism enhances cellular antiviral response to IFN-β. (**A**) PHGDH silencing reduces virus-induced IFN-β secretion. IFN-β secretion in CSFV-infected cells for 12 h and poly(I:C)-treated cells for 8 h was measured after PHGDH silencing. (**B**) PHGDH silencing decreases virus-induced IFN-β mRNA transcription. IFN-β mRNA levels in PK-15 cells stimulated by CSFV and poly(I:C) at different time points were assessed by qPCR after PHGDH silencing. (**C**) PHGDH enhances IFN-β secretion. IFN-β levels in PHGDH-overexpressing cells stimulated by CSFV and poly-IC were quantified by ELISA. (**D**) PHGDH elevates IFN-β mRNA transcription levels. IFN-β mRNA levels in PHGDH-overexpressing cells stimulated by CSFV and poly(I:C) at different time points were evaluated by qPCR. (**E and F**) PHGDH silencing decreases ISG15 and Mx1 mRNA levels. ISG15 (**E**) and Mx1 (**F**) mRNA levels in PK-15 cells stimulated by CSFV and poly(I:C) at different time points were measured by qPCR after PHGDH silencing. (**G and H**) PHGDH enhances ISG15 and Mx1 mRNA levels. ISG15 (**G**) and Mx1 (**H**) mRNA levels in PK-15 cells stimulated by CSFV and poly(I:C) at different time points were assessed by qPCR after PHGDH overexpression. (**I and J**) Serine deficiency impairs IFN-β secretion and mRNA transcription. IFN-β secretion and mRNA transcription after 6 h of exogenous serine treatment were evaluated by ELISA (**I**) and qPCR (**J**). (**K and L**) PHGDH-mediated IFN-β secretion and mRNA transcription levels reduced by K364 mutation. (**M and N**) IFN-β secretion and mRNA transcription levels inhibited by CBP-5884. PK-15 cells were treated with different concentrations of CBP-5884 for 24 h, and IFN-β secretion and mRNA transcription levels in the cells were quantified by ELISA (**M**) and qPCR (**N**). (**O**) IFN-β secretion levels decreased by PSAT1 or PSPH silencing. (**P**) IFN-β secretion levels lowered by PSAT1 or PSPH overexpression. Error bars indicate the mean (±SD) of three independent experiments. ^*^P < 0.05, ^**^P < 0.01, ^***^P < 0.001, and ^****^P < 0.0001 (*t*-tests or two-way ANOVA).

To investigate the role of PHGDH-mediated serine metabolism in antiviral immunity, we used specific small interfering RNA (siRNA) to knock down PHGDH in PK-15 cells stimulated by CSFV, or poly(I:C). It was found that IFN-β secretion and mRNA expression were significantly reduced in PHGDH-knockdown cells (Fig. 9A and B). In contrast, the overexpression of PHGDH in PK-15 cells increased IFN-β secretion and mRNA expression when stimulated by CSFV, or poly(I:C) (Fig. 9C and D). We also investigated the effect of PHGDH on IFN-β downstream genes and found that siPHGDH-treated cells had lower ISG15 and Mx1 mRNA levels than wild-type cells in response to CSFV, or poly(I:C) (Fig. 9E and F). In contrast, PHGDH overexpression enhanced ISG15 and Mx1 mRNA levels in CSFV, or poly(I:C)-stimulated cells (Fig. 9G and H). Serine metabolism is important for antiviral innate immunity upon virus infection (14), so we tested whether exogenous serine also affected antiviral innate immunity. We found that serine depletion reduced IFN-β secretion and mRNA transcription (Fig. 9I and J). PHGDH enzymatic activity is crucial for serine synthesis, so we explored whether PHGDH modulated antiviral innate immunity through its enzymatic activity. PHGDH-K364R mutants, which lack enzymatic activity, impaired the upregulation of IFN-β secretion and mRNA transcription, whereas PHGDH-WT did not (Fig. 9K and L), indicating that PHGDH enzymatic activity is required for IFN-β activation. Moreover, CBR-5884 (37), a selective small-molecule inhibitor of PHGDH enzymatic activity, also diminished IFN-β expression (Fig. 9M and N). PSAT1 and PSPH are key enzymes in the serine biosynthetic pathway, so we examined their role in antiviral innate immunity. We found that PSAT1-knockdown cells had lower IFN-β expression and PSAT1 overexpression cells had higher IFN-β expression (Fig. 9O and P). Similar results were obtained for PSPH knockdown and overexpression cells (Fig. 9O and P). These results suggest that PHGDH-mediated serine metabolism is involved in antiviral immunity.

### PHGDH enhances IFN-β signaling via mitochondria–MAVS–IRF3 axis

**Fig 10:**
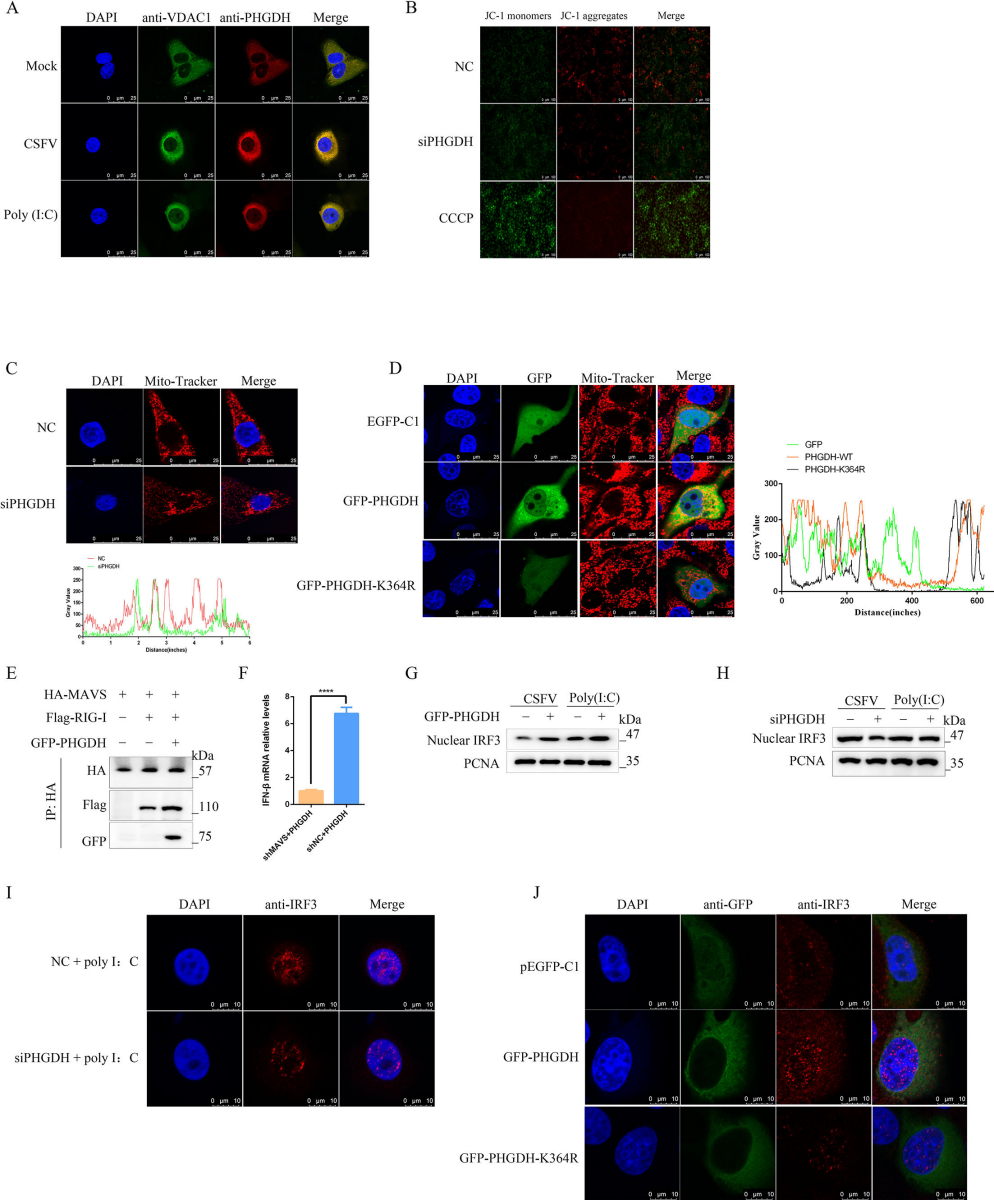
PHGDH enhances IFN-β signaling via the mitochondria–MAVS–IRF3 axis. (**A**) PHGDH localization to mitochondria induced by viral infection. PHGDH and mitochondrial membrane protein VDAC1 co-localization in CSFV- and poly(I:C)-stimulated cells was visualized by laser confocal microscopy. (**B**) PHGDH silencing impairs mitochondrial membrane potential. PHGDH-knockdown PK-15 cells were treated with JC-1. Mitochondrial membrane potential was assessed by the JC-1 fluorescent probe, and mitochondrial depolarization was determined by the ratio of red to green fluorescence intensity. (**C**) PHGDH silencing enhances mitochondrial division. PHGDH-inhibited cells showed increased mitochondrial length and division by laser confocal microscopy. (**D**) PHGDH overexpression augments the mitochondrial number. Mitochondrial morphological changes induced by PHGDH and its mutants were observed by confocal laser microscopy. (**E**) PK-15 cells were transfected with the indicated plasmid for 24 h. co-IP and western blot analysis with specified antibodies. (**F**) PHGDH-mediated IFN-β secretion reduced by MAVS knockout. Error bars indicate the mean (±SD) of three independent experiments. ^****^P < 0.0001 (*t*-tests). (**G and H**) IRF3 expression in the nucleus of PHGDH-silenced or overexpressing PK-15 cells was measured by karyoplasmic separation. (**I**) PHGDH silencing prevents IRF3 nuclear translocation. IRF3 localization in PHGDH-silent cells was visualized by confocal laser microscopy. (**J**) PHGDH overexpression facilitates IRF3 nuclear translocation. IRF3 localization in PHGDH-overexpressing or mutant PK-15 cells was visualized by confocal laser microscopy.

We hypothesized that PHGDH, which mediates IFN-β production, is linked to mitochondria, which are crucial for sensing amino acid deprivation and balancing energy metabolism and amino acid synthesis, as well as for antiviral resistance (38, 39). We detected the co-localization of PHGDH with the mitochondrial outer membrane protein VDAC1 in PK-15 cells stimulated with CSFV, or poly(I:C) (Fig. 10A). Consistent with our results, PHGDH translocate to mitochondria and stimulate mitochondrial translation and tumor progression in liver cancer (40). We used the Mito Tracker Red fluorescent probe and JC-1 to examine the effect of PHGDH on the mitochondrial structure and membrane potential in PHGDH-knockdown PK-15 cells. We measured the mitochondrial membrane potential using the JC-1 fluorescent probe and assessed the degree of mitochondrial depolarization by the ratio of red to green fluorescence intensity. PHGDH-inhibited cells showed a significant reduction in mitochondrial membrane potential, as shown in Fig. 10B. Laser confocal microscopy images showed that the mitochondrial length became longer and the division increased, upon labeling with the Mito Tracker Red fluorescent probe, in PHGDH-inhibited cells (Fig. 10C). In contrast, PHGDH overexpression shortened the mitochondria and increased their number, and this effect was blocked by the K364 mutant (Fig. 10D). Glycolysis remodeling has been reported to support viral replication by sequestering MAVS from RIG-I through a ternary complex with hexokinase (41) . PHGDH catalyzes the first rate-limiting step in the conversion of glycolysis-derived 3-phosphoglyceric acid to serine. To investigate whether PHGDH affects MAVS, we performed competitive co-IP experiments and showed that PHGDH enhanced the interaction between MAVS and RIG-I by binding to MAVS (Fig. 10E). We have previously shown that CSFV inhibits type I IFN secretion by blocking MAVS signaling (41), a mechanism also used by influenza A virus (42). To determine the role of RLR signaling in interferon secretion during CSFV infection, we used shRNA to knock down endogenous MAVS protein specifically. PK-15 cells transfected with shMAVS had a significant decrease in the mRNA expression of IFN genes compared with PHGDH-overexpressing cells transfected with scrambled shRNAs, as illustrated in Fig. 10F. Studies have shown that DNA virus, RNA virus, TLR3, and TLR4 promote IFN-β production because all these involve signaling pathways that converge at the node of TBK1 and IRF3 (43). Subsequently, we examined the translocation of the key transcription factor IRF3, which is essential for its functional exertion (44). The findings demonstrate that overexpression of PHGDH can enhance the protein expression of IRF3 in the nucleus, whereas silencing PHGDH reduces the nuclear protein expression of IRF3 (Fig. 10G and H). Moreover, PHGDH-knockdown PK-15 cells stimulated with poly(I:C) showed less nuclear translocation of IRF3 than host cells (Fig. 10I). Consistent with this, PHGDH-overexpressing PK-15 cells had more nuclear translocation of IRF3 than host cells, and this effect was suppressed by the K364 mutant (Fig. 10J). These data indicate that PHGDH deficiency prevents IFN-β production by inhibiting the mitochondria–MAVS–IRF3 axis.

### PHGDH-mediated serine metabolism inhibits viral proliferation

**Fig 11:**
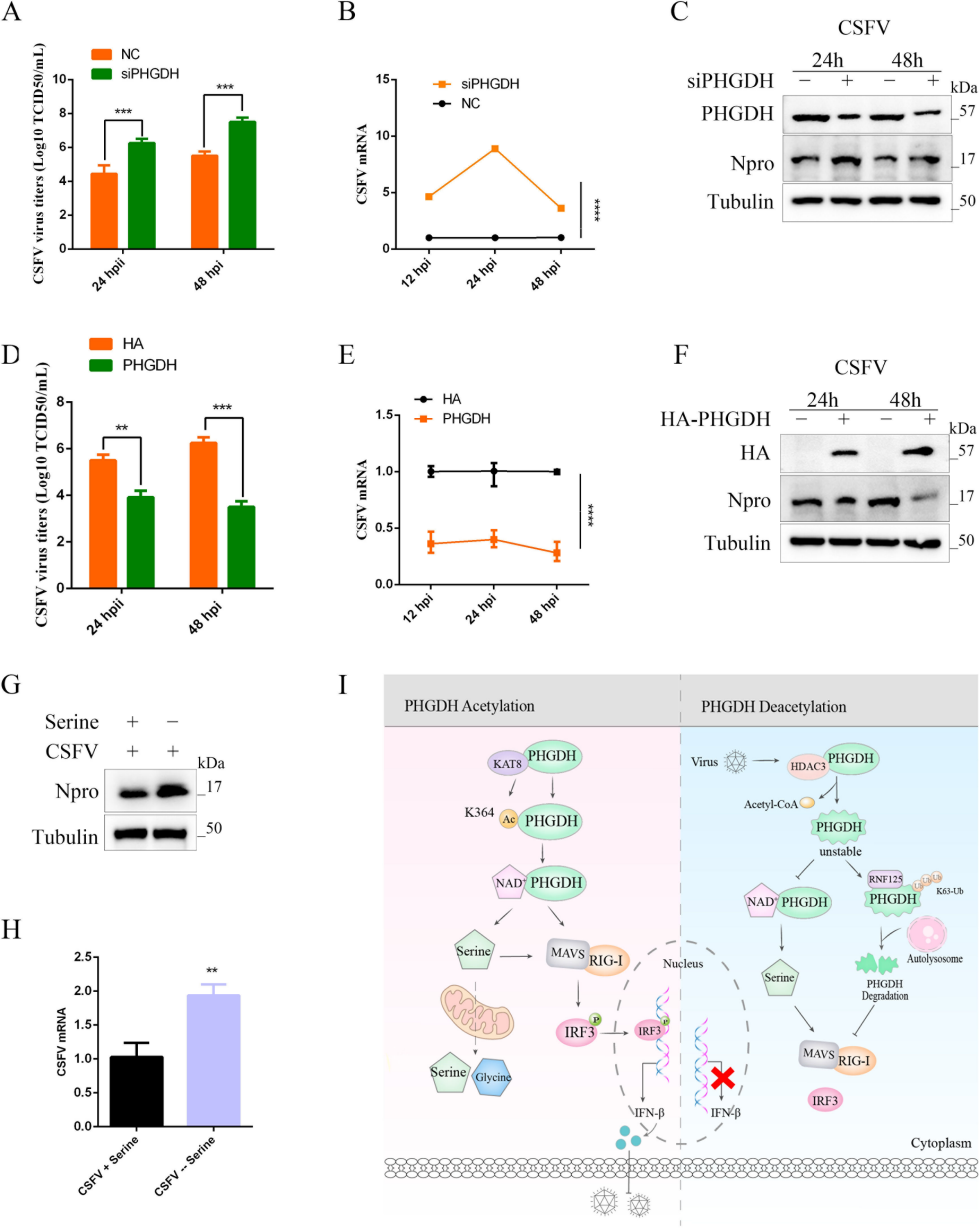
PHGDH-mediated serine metabolism inhibits viral proliferation. (**A, B, and C**) PHGDH silencing enhances CSFV replication. CSFV titer (**A**), mRNA (**B**), and viral protein Npro (**C**) in PHGDH-silenced cells were measured by IFA, qPCR, and western blotting. (**D, E, and F**) PHGDH overexpression suppresses CSFV replication. CSFV titer (**D**), mRNA (**E**), and viral protein Npro (**F**) in PHGDH-overexpressing cells were assessed at different time points. (**G**) Effect of exogenous deficiency of serine on CSFV protein expression. (**H**) Effect of exogenous deficiency of serine on CSFV copy numbers. (**I**) Molecular mechanisms by which CSFV infections modulate immunity to serine metabolism. Error bars indicate the mean (±SD) of three independent experiments. ^**^P < 0.01, ^***^P < 0.001, and ^****^P < 0.0001 (*t*-tests or two-way ANOVA).

We further investigated the antiviral ability of PHGDH in PK-15 cells. Knockdown of PHGDH can promote the replication of CSFV, decrease the titer of virus, and inhibit the mRNA level and protein expression of virus (Fig. 11A through C). Conversely, PHGDH overexpression reduced genome copy numbers, mRNA, and viral protein expression of CSFV as measured by the immunofluorescence assay (IFA) assay, qPCR, and western blotting (Fig. 11D through F). Further exploration of the effects of serine metabolism on viral replication revealed that serine deficiency promotes CSFV protein expression and increases the viral copy number (Fig. 11G and H). These results suggest that PHGDH-mediated serine metabolism suppresses viral proliferation. The above results suggest that viral infection modifies PHGDH by deacetylation, which in turn inhibits serine metabolism-mediated antiviral immunity (Fig. 11I).

## DISCUSSION

PHGDH is overexpressed in tumor cells and correlates with their aggressiveness and metastasis (18, 19, 45, 46). Recent studies have implicated PHGDH-mediated serine metabolism in antiviral innate immunity (14). However, the expression and function of PHGDH in viral infections remain unclear. We show that CSFV infection downregulates PHGDH expression, which is consistent with previous findings (14). One study reported that Parkin ubiquitinates and degrades PHGDH via the proteasome, thus inhibiting serine synthesis and tumorigenesis (18). Conversely, another study found that the monoubiquitination of PHGDH does not mediate its degradation but promotes its activity, facilitating colorectal cancer metastasis (19). Lysine acetylation can modulate the ubiquitination and lysosomal degradation of some metabolic enzymes (20, 47). We demonstrate that KAT8 and HDAC3 mediate the acetylation/deacetylation of the serine metabolizing enzyme PHGDH and that lysine acetylation regulates PHGDH activity and protein stability. Lysine deacetylation not only impairs PHGDH enzyme activity but also induces PHGDH degradation through K63-linked ubiquitin-selective autophagy. We reveal that deacetylation at K364 disrupts PHGDH enzyme activity and reduces its affinity for the substrates 3-phosphoglycerate and NAD. Additionally, we show that deacetylated PHGDH inhibits the *de novo* synthesis of serine and decreases SAM secretion, thereby suppressing serine metabolism-mediated antiviral innate immunity. These results uncover the effects of lysine acetylation on metabolic enzymes that regulate metabolic processes.

This study demonstrates that PHGDH-K364-Ac undergoes K63-linked ubiquitin-lysosomal degradation. PHGDH protein expression increased in PK-15 cells treated with the HDAC inhibitor TSA, which blocked the autophagic degradation of both endogenous PHGDH and exogenous PHGDH-K364R. Moreover, K364 deacetylation mediated the K63 ubiquitination of PHGDH. RNF125 is a RING motif-containing ubiquitin E3 ligase. It has been reported that RNF125 ubiquitinates substrates involved in innate and adaptive immunity, such as RNF125 mediating K48 ubiquitination of RIG-I and K63 ubiquitination of NLRP3 (48, 49). We demonstrated that PHGDH is a novel substrate for RNF125 that enhances the ubiquitination of PHGDH-K364R. E3 ubiquitination ligases mark proteins for ubiquitination, leading to their degradation by the autophagy–lysosome system (50). We identify that autophagy receptors p62 and NDP52 bind to PHGDH and mediate its degradation. These results uncover the key role of deacetylation and ubiquitination in regulating PHGDH protein stability.

More and more evidence suggests that metabolic enzymes are implicated in innate immunity (51–54). PHGDH, the rate-limiting enzyme in serine synthesis, has been shown to participate in antiviral innate immunity (14). However, we observed an inhibition of antiviral innate immunity upon the exogenous addition of serine. Furthermore, IFN-β production in PK-15 cells increased by PHGDH overexpression, while PHGDH silencing reduced IFN-β production. We found that virus infection induced the localization of PHGDH to mitochondria. In line with our findings, in liver cancer, PHGDH relocates to mitochondria and stimulates mitochondrial translation and tumor progression (40). We demonstrate that PHGDH silencing or deacetylation impairs mitochondrial function. PHGDH activates the RLR signaling pathway and augments the interaction between MAVS and RIG-I, facilitating IRF3 nuclear translocation. These results suggest that PHGDH may act as a key antiviral protein.

We demonstrate that viral infection downregulates PHGDH expression and serine metabolism. Virus infection increased HDAC3 expression in PK-15 cells, which inversely correlated with KAT8 and mediated PHGDH deacetylation. PHGDH enzyme activity and serine and glycine levels declined in virus-infected PK-15 cells. We also demonstrate that PHGDH suppresses viral replication. We reveal that viral infection impairs serine metabolism by enhancing PHGDH deacetylation, which facilitates viral replication. These results suggest that PHGDH modulates innate immunity.

## MATERIALS AND METHODS

### Serine and glycine analysis

0.1 mL methanol was added into 5 × 10^7^ cells and lysed in liquid nitrogen for three cycles of freezing and thawing and centrifuged at 5,000 × *g* for 10 min, and the supernatant was collected. The collected supernatant was analyzed by Hitachi L-2000 HPLC. Derivatized samples were separated on a C18 analytical column and eluted with a methanol/sodium carbonate gradient at 0.35 mL/min for the elution pump and 0.30 mL/min for the derivatization pump. The excitation and emission wavelengths were 340 and 450 nm, respectively.

### Mass spectrometry analysis

After reduction and alkylation, the test material was added with a specific amount of enzyme (mass ratio 1:50) and enzymatically digested at 37°C for 20 h. The enzyme digestion product was desalted by dialysis or ion exchange chromatography and lyophilized, redissolved in 0.1% FA solution, and stored at −20°C for use. Solvent A is an aqueous solution of 0.1% formic acid, and solvent B is an aqueous solution of 0.1% formic acid in acetonitrile (acetonitrile is 84%). After the column was equilibrated with 95% of liquid A, the samples were loaded onto the Trap column by an autosampler. The supernatant was separated by chromatography and analyzed by mass spectrometry. The detection mode was positive ion, the scanning range of the precursor ion was 300–1,800 m/z, the resolution of primary mass spectrometry was 70,000 at 200 m/z, the automatic gain control target was 1*e*6, the maximum IT was 50 ms, and the dynamic exclusion time (dynamic exclusion) was 30.0%. The mass spectra of peptides and peptide fragments were collected as follows: 20 fragmentation profiles were collected after each full scan (MS2 scan), the MS2 activation type was HCD, the isolation window was 2 m/z, the MS2 resolution was 17,500 at 200 m/z, the normalized collision energy was 27 eV, and the underfill was 0.1%.

### PHGDH activity assay

The PHGDH activity assay kit (colorimetry) (#ab273328, Abcam) was used to measure PHGDH activity in cells. The experiments were carried out according to the manufacturer’s instructions. In this assay, PHGDH converts 3-phosphoglycerate and NAD into 3-phosphohydroxyglerate and NADH. The oxidation of NADH reduces a colorimetric probe (e.g., WST-1) generating a strong, stable absorbance signal [optical density (OD): 450 nm]. Typical test mixtures in 96-well violet plates include PHGDH analysis buffer, PHGDH developer, PHGDH substrate, 1.25 mM NADH, and 0.5 U/mL PHGDH. Reaction rates were monitored by NADH oxidation and measured by absorbance using a plate reader (BioTek, Synergy HT) at 37°C, 340 nm.

### Immunoprecipitation

For immunoprecipitation, cells were lysed with 1% Nonidet P40 buffer containing 50 mM Tris-HCl (pH 7.5), 150 mM NaCl, 1 mM phenylmethanesulfonyl fluoride (PMSF), 2.5 µM TSA, and 25 mM NAM. The cell lysate was incubated overnight with the specified primary antibody at 4°C. The mixture was immunoprecipitated with protein A/G agarose beads (Beyotime, P2051) for 4 h, followed by washing three times in NP40 buffer and collection for immunoblot analysis.

### Plasmids and transfection

Expression plasmids for serine metabolizing enzyme (PHGDH, PSAT1, and PSPH), autophagy receptor (p62, NDP52, TAXBP1, and NBR1), acetyltransferases (GCN, CBP, KAT8, and PCAF), and deacetylases (HDAC1, HDAC2, HDAC3, HDAC4, HDAC5, HDAC7, and HDAC8) were cloned using conventional PCR. The Flag-KAT8(Δ1–121aa), Flag-KAT8(Δ121–232aa), Flag-KAT8(Δ232–458aa), GFP-PHGDH(Δ1–100aa), GFP-PHGDH(Δ101–200aa), GFP-PHGDH(Δ201–300aa), GFP-PHGDH(Δ301–400aa), and GFP-PHGDH(Δ401–533aa) were mutated from Flag-KAT8 and GFP-PHGDH, respectively. PHGDH enzymatically inactive mutants were generated by site-directed mutagenesis (Vazyme, C214) from GFP-PHGDH. The sequences of siRNAs targeting swine PHGDH, PSAT1, PSPH, HDAC3, KAT8, NDP52, and p62 were synthesized from Sangon Biotech and are listed as follows:

siPHGDH #1: 5′-GAUGAAGACUAUAGGGUAUTT-3′

siPHGDH #2: 5′-GAACAGAGCUGAACGGAAATT-3′

siPHGDH #3: 5′-GCACCUUUGCCCUUUGCAATT-3′

siKAT8 #1: 5′-GCAAGCACGAUGAGAUCAATT-3′

siKAT8 #2: 5′-CCACAAGACGCUGUACUUUTT-3′

siKAT8 #3: 5′-GGAAGUUCCUCAUCGCGUUTT-3′

siHDAC3 #1: 5′-GCUUCCACUCUGAGGACUATT-3′

siHDAC3 #2: 5′-GCAUCUCUGCAAGGAGCAATT-3′

siHDAC3 #3: 5′-GCAUCGAUGACCAGAGUUATT-3′

Lipofectamine 3000 (Thermo Fisher, L3000015) was used to transfect plasmids and siRNA when the cells were grown in 12-well plates at a density of 70%–80%.

### Virus infection

PK-15 and 3D4/2 cells were cultured to approximately 70% conﬂuence in cell culture plates and then infected with 1 multiplicity of infection (MOI) of CSFV at 37°C within a humidiﬁed 5% CO_2_ environment for 2 h. The viral inoculum was discarded, and the cells were washed twice with phosphate buffered saline (PBS). Next, the cells were incubated in Dulbecco's Modified Eagle's Medium (DMEM) containing 2% Fetal bovine serum (FBS) at 37°C with 5% CO_2_ for diﬀerent time points until harvesting.

### Protein half-life assay

For PHGDH half-life determination, PK-15 cells were treated with CHX (20 µM) for a specified time before collection.

### Metabolome analysis

For metabolite analysis, porcine alveolar macrophages (PAMs) from six lungs of pigs in each group were used for ultraperformance liquid chromatography-tandem time-of-flight mass spectrometry instrument analysis. Sixty milligrams of the sample was added to pre-cooled methanol/acetonitrile/aqueous solution (2:2:1, vol/vol); the following actions were performed: vortexing of the mixture, low-temperature ultrasound for 30 min, standing at −20°C for 10 min, centrifugation at 14,000 × *g* at 4°C for 20 min, vacuum drying of the supernatant, and addition of 100-µL acetonitrile aqueous solution (acetonitrile:water = 1:1, vol/vol), which was redissolved, vortexed, and centrifuged at 14,000 × *g* at 4°C for 15 min, and the supernatant was taken for sample analysis. After separation with an Agilent 1290 Infinity LC ultraperformance liquid chromatography system, the samples were analyzed by mass spectrometry with a Triple TOF 6600 mass spectrometer (AB SCIEX). Electrospray ionization positive and negative ion modes were used for detection.

The original data in Wiff format were converted into mzXML format by ProteoWizard, and then, XCMS software was used for peak alignment, retention time correction, and peak area extraction. Firstly, metabolite structure identification and data pre-processing were carried out for the data extracted from XCMS, and the quality of experimental data was evaluated. Finally, data analysis was carried out. The Human Metabolome Database (http://www.hmdb.ca/) and the METLIN Metabolite Database (METLIN; https://metlin.scripps.edu/index.php) are used to identify metabolites. SIMCA V13.0 (Swedish Umetrics) was used to implement multivariate statistical analyses, including principal component analysis and orthogonal partial least squares discrimination analysis (OPLS-DA). Differential metabolites were selected (OPLS-DA VIP >1, fold change >1.5 or <0.67, and *P* < 0.05). In addition, KEGG (http://www.genome.jp/kegg/) and MetaboAnalyst (http://www.metaboanalyst.ca/) were used to find new ways to metabolites.

### RNA-seq analysis

Total RNA was extracted from the PAMs using the TRIzol Reagent according to the manufacturer’s instructions (Magen). RNA samples were detected based on the A260/A280 absorbance ratio with a Nanodrop ND-2000 system (Thermo Scientific, USA), and the RIN of RNA was determined by an Agilent Bioanalyzer 4150 system (Agilent Technologies, California, USA). Only qualified samples will be used for library construction.

Paired-end libraries were prepared using a ABclonal mRNA-seq Lib Prep Kit (ABclonal, China) following the manufacturer’s instructions. The mRNA was purified from 1 µg total RNA using oligo (dT) magnetic beads followed by fragmentation carried out using divalent cations at elevated temperatures in ABclonal First Strand Synthesis Reaction Buffer. Subsequently, first-strand complementary DNAs (cDNAs) were synthesized with random hexamer primers and Reverse Transcriptase (RNase H) using mRNA fragments as templates, followed by second-strand cDNA synthesis using DNA polymerase I, RNAseH, buffer, and dNTPs. The synthesized double-stranded cDNA fragments were then adapter-ligated for the preparation of the paired-end library. Adaptor-ligated cDNAs were used for PCR amplification. PCR products were purified (AMPure XP system), and library quality was assessed on an Agilent Bioanalyzer 4150 system. Finally, sequencing was performed with an Illumina Novaseq 6000/MGISEQ-T7 instrument.

The data generated from the Illumina/BGI platform were used for bioinformatics analysis. All of the analyses were performed using an in-house pipeline from Shanghai Applied Protein Technology. The major software and parameters are as follows.

### Immunoblot analysis

Cells were lysed in RIPA lysis buffer (Beyotime, P0013B) containing 1 mM PMSF (Beyotime, ST506) for 20 min. The extracted proteins were quantified by the BCA protein assay kit (Beyotime, P0012) and boiled for 10 min in 5× SDS-PAGE sample loading buffer (Beyotime, P0015L). Equal protein samples were separated on 10% or 12% SDS-PAGE and transferred onto a polyvinylidene difluoride (PVDF) membrane. The PVDF membranes were first blocked in PBS containing 2% nonfat milk powder and 0.05% Tween 20 at 37°C for 1 h, which were then incubated with primary antibodies at 4°C overnight and then with the corresponding secondary antibodies conjugated to horseradish peroxidase (HRP) at 37°C for 2 h. The immunolabeled protein complexes were visualized using an ECL Plus kit (Beyotime, P0018), using the CanoScan LiDE 100 scanner system (Canon).

### Confocal immunofluorescence microscopy

Cells were grown in 35-mm Petri dishes (NEST, GBD-35-20) with a glass bottom. Cells were transfected with indicated plasmids and cultured for 24 h. Cells were washed with PBS and fixed with 4% paraformaldehyde (Sigma-Aldrich, P6148) for 30 min at room temperature and permeabilized with 0.2% Triton X-100 (Sigma-Aldrich, T8787) for 10 min. Cells were incubated with primary antibody in Primary Antibody Dilution Buffer at 4°C overnight. On the second day, after washing three times with PBST containing Tween 20, cells incubated with secondary antibody in PBS for 1 h at room temperature were rinsed with PBST and followed by staining of nuclei with 4',6-diamidino-2-phenylindole (DAPI) (Beyotime, C1002). Fluorescence signals were observed using a TCS SP2 confocal fluorescence microscope (Leica TCS SP8).

### Quantitative real-time RT-PCR

Cells were collected, and the total RNA was extracted using a total RNA Kit I (Omega, R6834-02). cDNA was synthesized using HiScript II Q RT SuperMix for qPCR (Vazyme, R223-01). Real-time qPCR was performed using ChamQ Universal SYBR qPCR Master Mix (Vazyme, Q711-02) using an iQ5 iCycler detection system (Bio-Rad, USA). Relative mRNA expression was assessed using the 2-ΔΔCt method and normalized to the housekeeping gene β-actin. The primer was synthesized by Sangon Biotech as follows:

PHGDH forward primer: GGGAGGAAATCGCCATCCAGTTC

Reverse primer: GCTTCAGCCAGACCAATCCAAGG

IFN-β forward primer: AGCAGATCTTCGGCATTCTC

Reverse primer: GTCATCCATCTGCCCATCAA

ISG15 forward primer: GCCCTTACCAGTGCCTTCTC

Reverse primer: GACAATGACTGCGGGGCTTA

Mx1 forward primer: GAACGAAGAAGACGAATGGAAGG

Reverse primer: GATGCCAGGAAGGTCTATGAGG

### Statistical analysis

The data are expressed as the mean ± standard deviation and were analyzed by *t*-tests (and nonparametric tests) using the GraphPad Prism 6 software. A value of *P* lesser than 0.05 was considered statistically significant.

## SUPPLEMENTAL MATERIAL

Fig. S1 mbio.02097-24-s0001.tif Association between CSFV protein and serine metabolizing enzyme.

Fig. S2 mbio.02097-24-s0002.tif Effect of CSFV infection on PHGDH enzyme activity.

Fig. S3 mbio.02097-24-s0003.tif PHGDH interacts with autophagy receptors.

Fig. S4 mbio.02097-24-s0004.tif Homology modeling of PHGDH-WT and PHGDH-Mut.

ASM does not own the copyrights to Supplemental Material that may be linked to, or accessed through, an article. The authors have granted ASM a non-exclusive, world-wide license to publish the Supplemental Material files. Please contact the corresponding author directly for reuse.
